# Exploring the aesthetic and sexual health impacts of reported lower extremity lymphedema-related symptoms in 174 patients following inguinal lymphadenectomy

**DOI:** 10.1016/j.jpra.2026.09.010

**Published:** 2026-09-12

**Authors:** Brett A. Hahn, Alieske Kleeven, Milan C. Richir, Arjen J. Witkamp, Anke M.J. Kuijpers, Kristien B.M.I Keymeulen, J. Henk Coert, Shan Shan Qiu, David D. Krijgh

**Affiliations:** a Department of Plastic and Reconstructive Surgery, University Medical Center Utrecht, Utrecht, the Netherlands; b Department of Plastic, Reconstructive and Hand Surgery, Maastricht University Medical Center, Maastricht, the Netherlands; c Department of Oncologic Surgery, University Medical Center Utrecht, Utrecht, the Netherlands; d Department of Surgical Oncology, The Netherlands Cancer Institute, Antoni van Leeuwenhoek Hospital, Amsterdam, the Netherlands; e Department of Surgery, Maastricht University Medical Center, Maastricht, the Netherlands

**Keywords:** Lower extremity lymphedema (LEL), Inguinal lymph node dissection (ILND), Inguinal lymphadenectomy, Quality of life, Lymphedema, Patient-reported outcome measures (PROMs)

## Abstract

**Background:**

Inguinal lymph node dissection (ILND) is associated with physical changes that extend beyond functional impairment. The psychosocial sequelae, particularly alterations in body image and sexual health, remain poorly characterized despite their profound impact on cancer survivors’ well-being. Understanding these outcomes is essential for comprehensive patient counseling and identifying candidates who may benefit from reconstructive interventions.

**Methods:**

This cross-sectional study was conducted and reported in accordance with the STROBE guidelines. Body image and sexual health outcomes were assessed in 174 patients who underwent ILND between 1990 and 2022 across three Dutch medical centers. Body image was assessed using the Body Image Scale (BIS), while sexual health was evaluated with the EORTC Sexual Health Questionnaire (SHQ-22). Clinical and surgical data were abstracted from medical records. Multivariate linear regression identified predictors of aesthetic and sexual health impairments.

**Results:**

Body image dissatisfaction and sexual health impairments were significantly more pronounced among patients with reported lymphedema-related symptoms. Patients who were female, of younger age, and who had postoperative complications including seroma requiring excision independently predicted worse body image outcomes. Female patients and those of older age were associated with reduced sexual function. Advanced malignancy stage for which surgery was indicated significantly increased sexual health-related morbidity.

**Conclusion:**

Body image disturbance and sexual dysfunction represent substantial yet under-recognized burdens following ILND. These findings underscore the need to consider psychosocial factors that may help identify high-risk patients who could benefit from interventions, such as reconstructive microsurgery.

## Introduction

Inguinal lymph node dissection (ILND) remains a cornerstone in the treatment of gynecologic, genitourinary, and dermatological malignancies with regional lymphatic spread.^1^ Beyond the well-documented functional sequelae of lower extremity lymphedema (LEL),^2^^,^^3^ the procedure’s impact on body image and sexual health represents a critical yet under-explored dimension of survivorship. For individuals navigating life after cancer treatment, alterations in physical appearance and sexual function can profoundly affect psychological well-being and interpersonal relationships.^4^^,^^5^

While meta-analyses estimate that 24% (95% CI: 17–31) of patients develop measurable lymphedema following ILND,^6^ the disconnect between objective measurements and subjective experience underscores the importance of patient-centered assessment of psychosocial outcomes.^7^^,^^8^ Although traditional approaches to LEL management have relied upon compression garments and manual lymphatic drainage, these interventions themselves have been documented to negatively impact body image and reinforce feelings of physical difference. The emergence of microsurgical reconstructive options such as lymphaticovenous anastomosis (LVA) and vascularized lymph node transfer (VLNT) creates new opportunities for comprehensive care.^9^

This study leverages a large, multicenter cohort to characterize body image and sexual health outcomes following ILND using validated patient-reported outcome measures (PROMs). By examining the relationship between lymphedema-related symptoms and psychosocial function, we aim to better understand their impact on patients’ quality of life. In addition, by identifying clinical predictors of aesthetic and sexual health impairments, we seek to provide evidence for more holistic patient counseling and to better inform selection criteria for emerging microsurgical interventions.

## Methods

### Study design and participants

This analysis utilized data from the Quality of life And Lower extremity edema after inguinal lymphadenectomY (QALY) cohort, comprising 174 patients who underwent ILND between 2009 and 2022 at three medical centers in the Netherlands: University Medical Center Utrecht (UMCU), the National Cancer Institute Antoni van Leeuwenhoek (AvL), and Maastricht University Medical Center (MUMC). Complete methodology regarding patient recruitment, inclusion criteria, and cohort characteristics has been described previously.^10^ Briefly, eligible patients were at least sixteen years old at time of consent, had undergone surgery at least one year prior to study enrollment, and were able to complete Dutch-language questionnaires which were distributed electronically. Patients in this cohort had not undergone conservative or surgical therapy for lymphedema.

### Data collection

Retrospective clinical data including demographic variables, tumor characteristics, surgical details, and postoperative complications were abstracted from electronic medical records as previously described.^10^ Follow-up duration was calculated from the date of ILND to completion of study questionnaires.

### Patient-reported outcome measures

All questionnaires were validated in Dutch populations with established psychometric properties.

### Lymphedema

The Lymphoedema Functioning, Disability and Health Questionnaire for Lower Limb Lymphoedema (Lymph-ICF-LL) was used to characterize patient-reported lymphedema-related symptoms.^11^ This 28-item instrument assesses impairments across five domains (physical function, mental function, general tasks/household activities, mobility activities, and life domains/social life) using an eleven-point scale (0-10). Total scores range from 0-100. For the purpose of this analysis, we applied an exploratory, symptom-based cut-off of >4 to distinguish patients reporting any degree of lymphedema-related symptom burden. This threshold has not been previously validated as a diagnostic criterion for lymphedema. Detailed scoring methodology and validation data have been reported previously.^11^

### Body image

Body image was assessed using the Body Image Scale (BIS), a 10-item questionnaire designed to evaluate appearance-related distress in cancer patients.^12^ Items span affective, cognitive, and behavioral domains, with response options ranging from 0 (“not at all”) to 3 (“very much”). Total scores range from 0 (minimal distress) to 30 (maximum distress), with higher scores indicating greater body image dissatisfaction.

### Sexual health

Sexual health was evaluated using the European Organization for Research and Treatment of Cancer 22-Item Sexual Health Questionnaire (EORTC SHQ-22), a validated instrument developed for cancer patients and survivors.^13^^,^^14^ The SHQ-22 comprises functional scales (sexual satisfaction, importance of sexual activity, libido) and symptom scales (sexual pain, fatigue, incontinence). Higher functional scale scores indicate better sexual health, while higher symptom scale scores reflect greater impairment. Gender-specific items assessed femininity and vaginal dryness (female participants) or masculinity and erectile confidence (male participants).

### Statistical analysis

Descriptive statistics summarized demographic, surgical, and PROM data. Between-group comparisons (patients who reported versus those who did not report lymphedema-related symptoms) utilized Pearson’s Chi-squared test for categorical variables and Welch’s t-test for continuous variables.

Multivariable linear regression models identified predictors of body image (BIS score) and sexual health outcomes (SHQ-22 functional and symptom scale scores). Independent variables included all demographic, clinical, surgical, and postoperative factors from medical records, plus the presence of lymphedema-related symptoms as determined by Lymph-ICF-LL. Sex, age, malignancy type, TNM stage, (neo)adjuvant treatment, laterality, treatment center, and follow-up duration were considered a priori confounders based on clinical and biological plausibility and were force-retained in all multivariable models regardless of statistical significance. Backward stepwise regression with Akaike Information Criterion (AIC) was subsequently applied to the remaining predictors to optimize model parsimony. Final models reported regression coefficients, 95% confidence intervals (CIs), and p-values. All analyses were conducted using R Statistical Software (v4.3.2).^15^ Statistical significance was set at p < 0.05 (two-sided).

### Ethical consideration

The University Medical Center Utrecht Institutional Review Board approved this study (#23-242/DB). All participants provided written informed consent prior to enrollment.

## Results

### Cohort characteristics

**Fig. 1:**
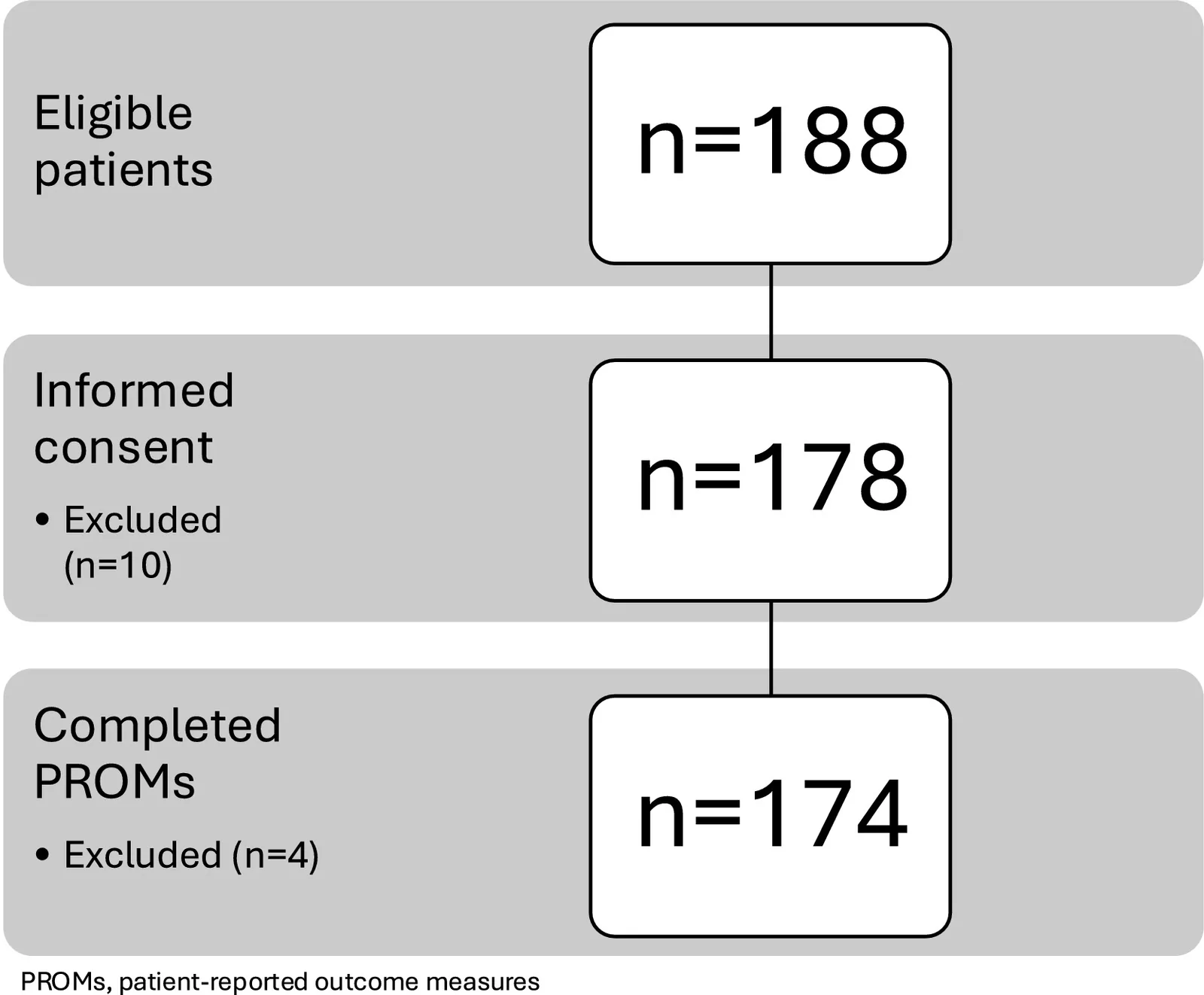
Patient flow diagram: eligibility, enrollment, and completion of patient-reported outcome measures (PROMs).

A total of 188 patients who met all inclusion criteria were contacted to participate in this study. Informed consent was obtained from 178 patients (95%). Four patients (2%) were lost to follow-up, and therefore were not included in the final analyses. A visual diagram can be found in Fig. 1. The cohort comprised 174 patients (47% female, 53% male) with a mean age of 58.99 ± 12.71 years at the time of ILND and 65.01 ± 12.51 years at follow-up. Patients underwent ILND between 2009-2022, with >75% of patients undergoing surgery after 2017 at a median year of 2019. The mean follow-up time was 72.23 ± 42.82 months. Complete demographic, clinical, and surgical characteristics have been reported previously,^10^ and are summarized in Table 1, Table 2.

**Table 1:** Comparison of participant health characteristics.

	**Total**	**Reported lymphedema-related symptoms, per patient**		
**Characteristic**	**N = 174***^1^*	**Yes**, N = 134*^1^*	**No**, N = 40*^1^*	**p-value***^2^*
*Demographics*				
Sex				<0.001
Female	82 (47%)	75 (56%)	7 (18%)	
Male	92 (53%)	59 (44%)	33 (83%)	
BMI, kg/m^2^	27.01 ± 4.69	27.06 ± 4.97	26.82 ± 3.69	0.91
Age at time of lymphadenectomy, years	58.99 ± 12.71	58.60 ± 12.61	60.29 ± 13.13	0.27
Age at time of follow-up, years	65.01 ± 12.52	64.70 ± 12.06	66.04 ± 14.06	0.30
Follow-up, months	72.23 ± 42.82	73.18 ± 43.46	69.05 ± 40.94	0.69
*Comorbidities*				
Diabetes				0.19
Yes	14 (8.0%)	13 (9.7%)	1 (2.5%)	
Hypertension				0.94
Yes	53 (30%)	41 (31%)	12 (30%)	
Cardiovascular disease				0.077
Yes	18 (10%)	17 (13%)	1 (2.5%)	
Rheumatoid arthritis				0.59
Yes	5 (2.9%)	5 (3.7%)	0 (0%)	
Inflammatory bowel disease				0.23
Yes	4 (2.3%)	2 (1.5%)	2 (5.0%)	
Thyroid disease				0.21
Yes	9 (5.2%)	5 (3.7%)	4 (10%)	
*Intoxications*				
Tobacco				0.77
Yes	17 (9.8%)	14 (10%)	3 (7.5%)	
Alcohol				0.058
Yes	113 (65%)	82 (61%)	31 (78%)	
Drugs				0.57
Yes	4 (2.3%)	4 (3.0%)	0 (0%)	
*Preoperative characteristics*				
Medical center				0.79
Antoni van Leeuwenhoek	159 (91%)	123 (92%)	36 (90%)	
Maastricht University Medical Center	8 (4.6%)	6 (4.5%)	2 (5.0%)	
Utrecht University Medical Center	7 (4.0%)	5 (3.7%)	2 (5.0%)	
Indication for lymphadenectomy				0.065
Angio sarcoma	1 (0.6%)	1 (0.7%)	0 (0%)	
Basosquamous carcinoma	1 (0.6%)	1 (0.7%)	0 (0%)	
Endometrium carcinoma	1 (0.6%)	0 (0%)	1 (2.5%)	
Melanoma	110 (63%)	90 (67%)	20 (50%)	
Merkel cell carcinoma	5 (2.9%)	5 (3.7%)	0 (0%)	
Myoepithelioma	1 (0.6%)	1 (0.7%)	0 (0%)	
Penile carcinoma	36 (21%)	21 (16%)	15 (38%)	
Prostate carcinoma	5 (2.9%)	3 (2.2%)	2 (5.0%)	
Squamous cell carcinoma	1 (0.6%)	1 (0.7%)	0 (0%)	
Testicular carcinoma	3 (1.7%)	3 (2.2%)	0 (0%)	
Urethral carcinoma	2 (1.1%)	1 (0.7%)	1 (2.5%)	
Vulvar carcinoma	8 (4.6%)	7 (5.2%)	1 (2.5%)	
TNM pathological stage				0.11
I	16 (9.2%)	13 (9.7%)	3 (7.5%)	
II	26 (15%)	15 (11%)	11 (28%)	
III	121 (70%)	97 (72%)	24 (60%)	
IV	11 (6.3%)	9 (6.7%)	2 (5.0%)	
Positive lymph nodes, preoperative				0.69
Yes	147 (84%)	114 (85%)	33 (83%)	
*Neoadjuvant therapy*				
Chemotherapy				0.32
Yes	5 (2.9%)	3 (2.2%)	2 (5.0%)	
Chemoradiotherapy				0.41
Yes	2 (1.1%)	1 (0.7%)	1 (2.5%)	
Immunotherapy				0.86
Yes	41 (24%)	32 (24%)	9 (23%)	
Radiotherapy				0.41
Yes	2 (1.1%)	1 (0.7%)	1 (2.5%)	
*Adjuvant therapy*				
Chemotherapy				>0.99
Yes	1 (0.6%)	1 (0.7%)	0 (0%)	
Chemoradiotherapy				>0.99
Yes	2 (1.1%)	2 (1.5%)	0 (0%)	
Hormone therapy				>0.99
Yes	1 (0.6%)	1 (0.7%)	0 (0%)	
Immunotherapy				0.41
Yes	48 (28%)	39 (29%)	9 (23%)	
Immuno-radiotherapy				0.20
Yes	8 (4.6%)	8 (6.0%)	0 (0%)	
Radiotherapy				0.83
Yes	28 (16%)	22 (16%)	6 (15%)	
*^1^*n (%); Mean ± SD				
*^2^*Fisher's exact test; Pearson's Chi-squared test; Wilcoxon rank sum test				
BMI, body mass index; TNM, tumor, nodes, metastases

**Table 2:** Comparison of lower extremity surgical characteristics.

	**Total**	**Reported lymphedema-related symptoms, per patient**		
**Characteristic**	**N = 174***^1^*	**Yes**, N = 134*^1^*	**No**, N = 40*^1^*	**p-value***^2^*
Lymphadenectomy laterality				0.13
Bilateral	18 (10%)	11 (8.2%)	7 (18%)	
Unilateral	156 (90%)	123 (92%)	33 (83%)	
Number of inguinal lymph nodes resected	9.67 ± 5.07	9.81 ± 5.29	9.18 ± 4.28	0.83
Number of inguinal lymph nodes with confirmed metastases	1.57 ± 2.57	1.76 ± 2.85	0.95 ± 1.01	0.086
Great saphenous vein surgical technique				0.90
Ligation	138 (79%)	106 (79%)	32 (80%)	
Sparing	36 (21%)	28 (21%)	8 (20%)	
Reconstruction during lymphadenectomy				
No	174 (100%)	134 (100%)	40 (100%)	
Preoperative antibiotic use				0.43
Yes	69 (40%)	51 (38%)	18 (45%)	
Postoperative antibiotic use				0.95
Yes	79 (45%)	61 (46%)	18 (45%)	
Wound dehiscence				0.89
Yes	55 (32%)	42 (31%)	13 (33%)	
Seroma development				0.66
Yes	118 (68%)	92 (69%)	26 (65%)	
Seroma requiring drainage				0.74
Yes	104 (60%)	81 (60%)	23 (58%)	
Wounds requiring ≥ 6 weeks therapy				0.71
Yes	74 (43%)	58 (43%)	16 (40%)	
Reoperations				>0.99
Yes	5 (2.9%)	4 (3.0%)	1 (2.5%)	
Other complications				0.50
None	115 (66%)	85 (63%)	30 (75%)	
SSI	50 (29%)	42 (31%)	8 (20%)	
Seroma cavity exploration	1 (0.6%)	1 (0.7%)	0 (0%)	
Hematoma exploration	1 (0.6%)	1 (0.7%)	0 (0%)	
Postoperative bleeding	2 (1.1%)	2 (1.5%)	0 (0%)	
Seroma excision	1 (0.6%)	1 (0.7%)	0 (0%)	
Abscess	4 (2.3%)	2 (1.5%)	2 (5.0%)	
*^1^*n (%); Mean ± SD				
*^2^*Fisher's exact test; Wilcoxon rank sum test; Pearson's Chi-squared test				
SSI, surgical site infection				

### Patient-reported outcome measures

#### Lymphedema

Using the Lymph-ICF-LL questionnaire, 134 patients (77%) reported lymphedema-related symptoms at a mean follow-up of 72.23 ± 42.82 months. Patients who reported lymphedema-related symptoms were characterized by substantially higher total Lymph-ICF-LL scores (26.01 ± 17.29 vs. 1.41 ± 1.37), reflecting the symptom-based threshold used to define group membership (Table 3.). The clinical relevance of this symptom burden is demonstrated through its independent associations with outcomes measured by separate validated instruments, including the Body Image Scale and EORTC sexual health scales, detailed below.

**Table 3:** Impairments in function, activity limitations, and participation restrictions reported by participants with the Lymph-ICF-LL questionnaire.

	**Reported lymphedema-related symptoms, per patient**		
**Lymph-ICF-LL Component**	**Yes**, N = 134*^1^*	**No**, N = 40*^1^*	**p-value***^2^*
***Physical function***			
Score	32.31 ± 19.81	2.67 ± 3.60	<0.001
Severity interpretation			<0.001
No problem	3 (2.2%)	31 (78%)	
Small problem	56 (42%)	9 (23%)	
Moderate problem	46 (34%)	0 (0%)	
Severe problem	29 (22%)	0 (0%)	
***Mental function***			
Score	19.79 ± 20.08	0.58 ± 1.87	<0.001
Severity interpretation			<0.001
No problem	33 (25%)	37 (93%)	
Small problem	54 (40%)	3 (7.5%)	
Moderate problem	32 (24%)	0 (0%)	
Severe problem	15 (11%)	0 (0%)	
***General tasks/household activities***			
Score	18.21 ± 22.55	0.08 ± 0.53	<0.001
Severity interpretation			<0.001
No problem	50 (37%)	40 (100%)	
Small problem	47 (35%)	0 (0%)	
Moderate problem	16 (12%)	0 (0%)	
Severe problem	21 (16%)	0 (0%)	
***Mobility***			
Score	29.17 ± 21.86	1.86 ± 3.36	<0.001
Severity interpretation			<0.001
No problem	12 (9.0%)	32 (80%)	
Small problem	54 (40%)	8 (20%)	
Moderate problem	39 (29%)	0 (0%)	
Severe problem	29 (22%)	0 (0%)	
***Life domains/social life***			
Score	27.24 ± 22.18	0.90 ± 2.09	<0.001
Severity interpretation			<0.001
No problem	20 (15%)	36 (90%)	
Small problem	53 (40%)	4 (10%)	
Moderate problem	35 (26%)	0 (0%)	
Severe problem	26 (19%)	0 (0%)	
***Total***			
Score	26.01 ± 17.29	1.41 ± 1.37	<0.001
Severity interpretation			<0.001
No problem	0 (0%)	40 (100%)	
Small problem	77 (57%)	0 (0%)	
Moderate problem	38 (28%)	0 (0%)	
Severe problem	19 (14%)	0 (0%)	
*^1^*Mean ± SD; n (%)			
*^2^*Wilcoxon rank sum test; Pearson's Chi-squared test; Fisher's exact test			
Lymph-ICF-LL, Lymphoedema Functioning Disability and Health Questionnaire for Lower Limb Lymphoedema			

#### Body image

Patients with lymphedema-related symptoms reported significantly greater body image dissatisfaction across all ten BIS items compared to those without symptoms (Table 4). Specific areas of concern included feeling self-conscious about appearance (1.15 ± 1.04 vs. 0.28 ± 0.56; p < 0.001), feeling less physically attractive (0.99 ± 1.08 vs. 0.13 ± 0.40; p < 0.001), and greater dissatisfaction with body appearance (1.04 ± 1.04 vs. 0.23 ± 0.48; p < 0.001). Behavioral changes were also evident, with patients with lymphedema-related symptoms reporting greater difficulty looking at their naked body (0.60 ± 0.92 vs. 0.10 ± 0.38; p = 0.001) and increased avoidance of others due to appearance concerns (0.37 ± 0.70 vs. 0.03 ± 0.16; p = 0.002). Overall, mean total BIS scores were substantially higher among those with lymphedema-related symptoms (7.43 ± 6.20 vs. 1.92 ± 2.68; p < 0.001), reflecting potential body image disturbance.

**Table 4:** Comparison of Body Image Scale scores among participants with self-reported lymphedema-related symptoms.

	**Reported lymphedema-related symptoms, per patient**		
**Characteristic**	**Yes**, N = 134*^1^*	**No**, N = 40*^1^*	**p-value***^2^*
1. Been feeling self-conscious about your appearance	0.79 ± 0.83	0.05 ± 0.22	<0.001
2. Felt less physically attractive as a result of your disease or treatment	0.96 ± 0.92	0.18 ± 0.38	<0.001
3. Been dissatisfied with your appearence when dressed	0.54 ± 0.72	0.08 ± 0.27	<0.001
4. Been feeling less feminine/masculine as a result of your disease or treatment	0.53 ± 0.87	0.18 ± 0.68	0.003
5. Find it difficult to look at yourself naked	0.69 ± 0.90	0.18 ± 0.45	<0.001
6. Been feeling less sexually attractive as a result of your disease or treatment	1.06 ± 0.99	0.30 ± 0.65	<0.001
7. Avoid people because of the way you felt about your appearance	0.23 ± 0.55	0.03 ± 0.16	0.015
8. Been feeling the treatment has left your body less whole	0.98 ± 0.93	0.28 ± 0.60	<0.001
9. Felt dissatisfied with your body	0.90 ± 0.86	0.35 ± 0.70	<0.001
10. Been dissatisfied with the appearance of your scar	0.76 ± 0.92	0.33 ± 0.68	0.007
BIS total score (out of 30)	7.43 ± 6.20	1.92 ± 2.68	<0.001
*^1^*Mean ± SD			
*^2^*Wilcoxon rank sum test			
BIS, Body Image Scale			

### Predictors of patient-reported outcomes

#### Body image predictors

Multivariable linear regression identified several independent predictors of body image dissatisfaction Table 5 (Table 6). The presence of lymphedema-related symptoms emerged as the strongest predictor, associated with a 4.55-point increase in BIS scores (95% CI: 2.55, 6.55; p < 0.001).

**Table 5:** Comparison of physical, psychological, and social effects on sexual health reported by participants with the EORTC Sexual Health Questionnaire.

	**Reported lymphedema-related symptoms, per patient**		
	**Yes**, N = 134*^1^*	**No**, N = 40*^1^*	**p-value***^2^*
**SHQ item**			
1. Importance of an active sex life	2.05 ± 0.90	2.08 ± 0.80	0.71
2. Had decreased libido	2.13 ± 1.07	1.53 ± 0.93	<0.001
3. Been satisfied with your level of sexual desire	2.22 ± 0.95	2.63 ± 1.00	0.025
4. Been satisfied with your sex life	2.12 ± 0.96	2.68 ± 0.89	0.001
5. Been worried about being incontinent (urine/stool)	1.49 ± 0.80	1.23 ± 0.58	0.034
6. Fatigue or a lack of energy have affected your sex life	1.71 ± 0.86	1.25 ± 0.59	<0.001
7. The treatment has affected your sexual activity	1.96 ± 1.10	1.68 ± 1.07	0.10
8. Been worried that sex would be painful	1.38 ± 0.79	1.08 ± 0.35	0.011
9. Had communication with health professionals about sexual issues	1.04 ± 0.23	1.08 ± 0.35	0.54
10. Been satisfied with the communication about sexual issues between yourself and your partner	1.66 ± 1.64	2.25 ± 1.78	0.041
11. Been worried that your partner may cause you pain during sexual contact	0.82 ± 0.84	0.78 ± 0.58	0.83
12. Been satisfied with your level of intimacy	1.87 ± 1.50	2.13 ± 1.59	0.33
13. Felt insecure regarding your ability to satisfy your partner	1.13 ± 1.23	0.93 ± 0.92	0.68
14. Confident about obtaining and maintaining an erection when you had sex (men)	1.97 ± 1.10	2.36 ± 1.17	0.11
15. Felt less masculine as a result of your disease or treatment (men)	2.02 ± 1.22	1.45 ± 0.83	0.030
16. Felt less feminine as a result of your disease or treatment (women)	1.67 ± 0.81	1.00 ± 0.00	0.017
17. Been sexually active	1.69 ± 0.71	1.93 ± 0.73	0.072
18. Sexual activity has been enjoyable for you	2.82 ± 0.78	2.96 ± 0.69	0.43
19. Been satisfied with your ability to reach an orgasm	2.70 ± 0.86	3.07 ± 0.81	0.053
20. Felt pain during/after sexual activity	1.23 ± 0.45	1.00 ± 0.00	0.008
21. Felt sexual enjoyment	2.77 ± 0.73	3.11 ± 0.69	0.038
22. Experienced a dry vagina during sexual activity (women)	1.23 ± 1.29	1.14 ± 1.35	0.91
*Total scale scores*			
Functional	17.07 ± 8.66	22.03 ± 9.25	0.002
Symptoms	14.85 ± 5.12	11.80 ± 2.97	<0.001

*^1^*Mean ± SD

*^2^*Wilcoxon rank sum test

EORTC, European Organisation for Research and Treatment of Cancer; SHQ, Sexual Health Questionnaire

**Table 6:** Multivariate analyses of predictors of poorer Body Image Survey score in patients following inguinal lymphadenectomy.

		95% Confidence Interval		
Predictor	Estimate	Lower	Upper	p-value
Reported lymphedema-related symptoms: **Yes**	4.549	2.550	6.548	< 0.001
Sex: **Female**	2.459	0.679	4.239	0.007
Age at time of follow-up: **< 65**	1.936	0.273	3.600	0.023
Other postoperative complications: **Seroma excision**	11.928	1.571	22.285	0.024

Female patients reported significantly poorer body image compared to their male counterparts (Estimate: 2.46; 95% CI: 0.68, 4.24; p = 0.007). Patient age influenced body image outcomes, with those younger than 65 years at follow-up experiencing significantly greater body image disturbance (Estimate: 1.94; 95% CI: 0.27, 3.60; p = 0.023). Neither duration of follow-up had, nor stage or type of malignancy had an impact on body image outcomes.

While surgical factors did not play a significant role, postoperative complications predicted greater dissatisfaction in body image: seroma requiring excision was associated with substantially elevated BIS scores (Estimate: 11.93; 95% CI: 1.57, 22.29; p = 0.024).

#### Sexual health predictors

Predictors of sexual health outcomes varied between functional and symptom dimensions (Table 7). For functional outcomes, lymphedema-related symptoms were associated with a 4.71-point decrease in functional scale scores (95% CI: -7.57, -1.86; p = 0.001), indicating reduced sexual function. Female patients experienced a 4.56-point decrease in functional sexual health scores compared to males (95% CI: -7.17, -1.96; p = 0.001). Older age (≥65 years) was also predictive of lower sexual function at both time of surgery (Estimate: -3.43; 95% CI: -6.75, -0.12 p = 0.043), as was well as time of follow-up (Estimate: -5.80; 95% CI: -9.05, -2.55; p = 0.001).

**Table 7:** Multivariate analyses of predictors of poorer EORTC Sexual Health Questionnaire functional and symptoms scale scores in patients following inguinal lymphadenectomy.

		95% Confidence Interval		
Predictor	Estimate	Lower	Upper	p-value
SHQ functional scale score				
Reported lymphedema-related symptoms: **Yes**	-4.714	-7.569	-1.860	0.001
Sex: **Female**	-4.564	-7.172	-1.956	0.001
Age at time of surgery: **≥ 65**	-3.432	-6.747	-0.117	0.043
Age at time of follow-up: **≥ 65**	-5.797	-9.045	-2.550	0.001
Intoxications: **Smoking**	-6.235	-10.077	-2.392	0.002
SHQ symptom scale score				
Reported lymphedema-related symptoms: **Yes**	3.328	1.717	4.940	< 0.001
TNM Stage: **II**	5.551	2.699	8.402	<0.001
TNM Stage: **III**	3.615	1.269	5.962	0.003
TNM Stage: **IV**	4.275	0.841	7.709	0.015
EORTC, European Organisation for Research and Treatment of Cancer; SHQ, Sexual Health Questionnaire; TNM, tumor, nodes, metastases. Higher scores in the functional scale score indicate better functional level whereas higher scores in the symptoms scale score indicate greater severity of the symptoms.				

For symptom-related morbidity, lymphedema-related symptoms predicted a 3.33-point increase in symptom scale scores (95% CI: 1.72, 4.94; p < 0.001). Advanced malignancy stages were independently associated with greater sexual health symptoms: TNM stage II (Estimate: 5.55; 95% CI: 2.70, 8.40; p < 0.001), stage III (Estimate: 3.62; 95% CI: 1.27, 5.96; p = 0.003), and stage IV (Estimate: 4.28; 95% CI: 0.84, 7.71; p = 0.015) all predicted higher symptom burden compared to stage I. The type of malignancy for which patients underwent ILND, however, did not prove to be a significant predictor of sexual health symptoms. Treatment-related factors, such as the (neo)adjuvant therapy for which patients received in addition to ILND did not influence sexual health morbidity.

## Discussion

This study demonstrates that reported lymphedema-related symptom burden following ILND, as defined by the Lymph-ICF-LL, is independently associated with clinically significant impairments in body image and sexual health as measured by separate validated instruments. Among 174 patients, those who reported lymphedema-related symptoms demonstrated greater body image dissatisfaction and multidimensional sexual health impairments across independent outcome measures that persisted years after surgery. The cross-instrument nature of these associations, linking a lymphedema-specific measure to distinct body image and sexual health scales, strengthens the evidence that symptom burden following ILND carries meaningful psychosocial consequences beyond the physical domain. While the relative contributions of the underlying malignancy, concurrent treatments, and ILND itself cannot be disentangled without a non-ILND comparator cohort, these findings highlight the breadth of morbidity in this population. Furthermore, these findings challenge the traditional focus on objective limb circumference and volume measurements, highlighting the need for patient-centered outcome assessment in surgical oncology.

The relationship between reported lymphedema-related symptoms and body image disturbance aligns with cancer survivorship literature demonstrating that visible physical changes profoundly affect self-perception.^16^, ^17^, ^18^ Our findings extend this understanding by quantifying specific domains of concern. Patients who reported lymphedema-related symptoms did not only feel less physically attractive and more self-conscious, but also exhibited behavioral changes that included social avoidance and difficulty viewing their own bodies. These responses suggest that body image disturbance represents more than aesthetic concern, but also reflects fundamental disruption to bodily integrity and social identity.^19^

The sexual health findings reveal a complex interplay between physical symptoms, psychological factors, and intimate function. Despite maintaining similar values regarding sexual activity importance and comparable engagement levels, patients with lymphedema-related symptoms derived significantly less satisfaction and enjoyment. This disconnect suggests that physical limitations such as pain, fatigue, and mobility restrictions, create barriers to pleasurable sexual expression rather than eliminating sexual interest.

The predictors identified in this study may offer useful insights for clinical practice. Patient-reported lymphedema-related symptoms appeared to be the most consistent correlate of morbidity, associated with worse body image as well as both reduced sexual function and greater sexual symptom burden. This consistency suggests that patients’ reported symptom burden could be an important determinant of quality of life after ILND. Sex and age associations appeared to diverge by outcome: female sex was linked to worse body image and sexual function alike, while younger age was associated with greater body image dissatisfaction and older age with poorer sexual function. As causal mechanisms cannot be inferred from these data, this pattern may reflect distinct, age- and sex-related pathways linking ILND to psychosocial and sexual morbidity, though this remains to be further investigated. Advanced malignancy stage was associated with greater sexual symptom burden, which could reflect the combined influence of treatment intensity and psychological distress. Notably, seroma requiring excision was associated with a marked increase in body image disturbance, suggesting that visible scarring or prolonged wound healing may compound the psychological burden of lymphedema itself

Conservative management approaches for lymphedema may paradoxically exacerbate psychosocial concerns. Compression garments, while effective for symptom control, are visible markers of physical difference that may reinforce body image disturbance and limit clothing choices.^20^, ^21^, ^22^ Despite being aware of the risk of exacerbation of lymphedema-related symptoms, patients have reported to forgo wearing compression garments in favor of clothing that makes them feel more attractive and regain a sense of control over their own bodies.^23^ Ultimately, the burden of conservative approaches, combined with their imperfect efficacy, creates situations where patients must choose between symptom management and psychosocial well-being.

These findings suggest that microsurgical interventions may have a role in addressing both the functional and psychosocial dimensions documented in this study. This study, however, did not directly evaluate patients undergoing LVA or VLNT. Conclusions in this study are therefore based exclusively on patient-reported outcomes, which cannot substitute longitudinal prospective studies with objective outcomes. While more long-term research is needed to better understand which patients stand to benefit the most from LVA or VLNT, estimates of clinical improvement in extremity circumference and volume change range between 30-40%.^24^ Even in the absence of clinical improvement, patients who undergo reconstructive microsurgery in treatment of LEL have reported improved quality of life outcomes with greater response to conservative therapies and in some cases a reduction in the overall use of compression garments to manage symptoms.^25^^,^^26^

### Strengths and limitations

This multicenter study across three Dutch academic centers with 174 patients represents one of the largest cohorts examining psychosocial outcomes following ILND. The comprehensive PROMs encompassing validated measures of lymphedema symptoms, body image, and sexual health, provide a holistic view of survivorship extending beyond traditional clinical endpoints. Inclusion of diverse malignancy types reflects real-world clinical practice and allows assessment of disease-specific effects.

This study spans surgical procedures performed between 2009 and 2022, during which surgical technique, perioperative management, and oncological treatment protocols may have evolved. Notably, 75% of patients underwent surgery after 2017, with a median surgery year of 2019, indicating that the cohort is weighted toward more recent practice patterns; nonetheless, residual heterogeneity from the earlier years of the study period may have influenced lymphedema risk and patient-reported outcomes in ways not fully captured by our covariates. In addition, our cohort is subject to potential survival bias, as outcome data were necessarily collected from patients who survived to and completed follow-up assessment.

TNM stage was included as a covariate in the linear regression models employed in this study using each malignancy’s disease-specific staging criteria without harmonization across cancer types. Because melanoma accounted for 63% of the cohort’s indication for undergoing surgery, subgroup sizes for non-melanoma malignancies were too small to support a meaningful harmonized comparison. Comparisons of stage across the cohort should therefore be interpreted with caution, as equivalent numerical stages may not reflect comparable disease burden between different malignancies.

This study did not collect data surrounding adjacent conservative management methods, such as compression therapy or manual lymphatic drainage that patients may have undergone following ILND. All perioperative variables were ascertained from electronic medical records and, while such care is generally well-documented within the Dutch healthcare system due to referral requirements, any self-initiated or externally obtained care outside the documented pathway that could potentially influence patient-reported outcomes may not have been fully captured.

While the Lymph-ICF-LL is the first Dutch questionnaire with evidence of reliability and validity for assessing impairments in patients with secondary LEL, the scores capture symptom burden rather than objective lymphedema severity. Incorporation of imaging such as indocyanine green lymphangiography (ICG)^27^ may strengthen future investigations by distinguishing subjective symptoms from physiological lymphatic dysfunction, particularly when considering patients for microsurgical intervention. Objective measures of lymphedema severity, however, have previously been found to be incongruent with the physical and psychological impacts following lymph node dissection and therefore may not fully capture morbidity.^24^ The symptom-based approach in this study remains clinically relevant, as PROMs tend to closely reflect patients’ lived experiences more accurately perhaps than functional imaging.^28^

The substantially higher rate of reported lymphedema-related symptoms in this cohort (77%) compared with previously reported estimates of objectively measured lymphedema (24%)^6^ likely reflects fundamental differences in case ascertainment. Objective measures rely on circumferential or volumetric change and often require a minimum threshold to meet diagnostic criteria, whereas the Lymph-ICF-LL captures the broader, patient-perceived impact of even mild or intermittent symptoms across physical, mental, and social domains. This distinction is consistent with prior work demonstrating inconsistent and heterogeneous definitions of lymphedema across the literature,^6^ and it underscores a central premise of the current study: symptom burden, as experienced by patients, may not correspond directly to objective disease severity yet still carries meaningful psychosocial consequences.

## Conclusion

Body image disturbance and sexual health impairment represent substantial yet consistently under-recognized outcomes associated with lymphedema-related symptoms following ILND. The presence of patient-reported lymphedema-related symptoms strongly predict impaired psychosocial outcomes that contribute to morbidity. These findings advocate for expanded outcome measures that identify patients with lymphedema-related symptoms who are most vulnerable to body image concerns and sexual dysfunction. For patients at greatest risk of morbidity attributed to body image dissatisfaction and sexual health impairment as identified by linear regression models in this study, early consideration of reconstructive microsurgery may be something to consider between doctors and patients. Whether such microsurgical interventions offer meaningful improvements in both physical symptoms and psychosocial well-being remains to be established in future prospective studies.

## Funding

None.

## Ethical approval

Approved by the University Medical Center Utrecht Institutional Review Board (#23-242/DB).

## Declaration of competing interest

The authors declare that they have no known competing financial interests or personal relationships that could have appeared to influence the work reported in this paper.
